# Could excessive zinc supplementation during pregnancy cause menkes disease? A hypothesis worth investigating

**DOI:** 10.3389/fped.2026.1734361

**Published:** 2026-04-13

**Authors:** Uma Maheshwari Mugundan, Venkatesan Saravanan, Muhasaparur Ganesan Rajanandh

**Affiliations:** 1 Department of Pharmacy Practice, SRM College of Pharmacy, Faculty of Medicine and Health Sciences, SRM Institute of Science and Technology, Kattankulathur, Chengalpattu, Tamil Nadu, India; 2 School of Pharmacy, SBV Chennai, Sri Balaji Vidyapeeth (Deemed to be University), Pondicherry, India

**Keywords:** ATP7A, copper deficiency, environmental modifier, fetal development, menkes disease, neonatal health, pregnancy, zinc supplementation

## Abstract

**Background:**

Copper is an essential micronutrient critical for fetal neurodevelopment, haematopoiesis, angiogenesis, and immune function, with maternal transfer—particularly in the third trimester—playing a key role in establishing fetal copper stores. Disruption of this process, due to genetic defects or micronutrient imbalance, can lead to significant neonatal complications.

**Objective:**

This review examines the potential role of excessive maternal zinc supplementation as an underrecognized environmental modifier in Menkes disease (MD), an X-linked disorder caused by mutations in the ATP7A copper transporter. We hypothesize that in fetuses with ATP7A dysfunction, elevated maternal zinc intake may further impair copper absorption and placental transfer through competitive antagonism, thereby exacerbating fetal copper deficiency and influencing disease severity or onset.

**Evidence:**

Limited clinical data in pregnant women demonstrate that zinc supplementation can reduce maternal and fetal copper levels, supported by consistent findings from animal models and case reports indicating disrupted copper homeostasis. However, no large-scale or disease-specific studies have evaluated this interaction in relation to Menkes disease or neonatal outcomes.

**Conclusion:**

Given the widespread use of zinc supplementation, particularly during the COVID-19 era, its impact on fetal copper status in genetically susceptible populations warrants urgent investigation. Targeted retrospective analyses and well-designed prospective studies are needed to validate this hypothesis. A re-evaluation of prenatal micronutrient strategies with emphasis on trace element balance may improve risk stratification and optimize maternal–fetal health outcomes.

## Introduction

Menkes disease (MD) is a rare X-linked recessive neurodegenerative disorder caused by mutations in the ATP7A gene, which encodes a critical copper-transporting ATPase involved in cellular copper efflux and distribution. Disruption of this pathway leads to systemic copper deficiency, manifesting as progressive neurological deterioration, connective tissue abnormalities, and the characteristic brittle, depigmented (steel-coloured) hair in affected infants. Clinical symptoms typically emerge within the first few months of life, including hypotonia, seizures, failure to thrive, and developmental regression. Without early intervention, such as neonatal copper supplementation, most affected children do not survive beyond the age of three (1, 2).

The incidence of MD shows wide geographic variation. In the United States, it is estimated to occur in 1 in 50,000 to 1 in 250,000 live births, with about one-third of cases resulting from spontaneous (*de novo*) mutations (3). Among male live births, the condition affects approximately 1 in 35,000. A population-based study in Japan (1993–2003) reported a much lower overall incidence of 1 in 2.8 million live births and 4.9 cases per million male births. In contrast, data from Australia suggest a higher incidence, ranging from 1 in 50,000 to 1 in 100,000, possibly due to a founder effect. Although the disorder predominantly affects males, rare cases in females have been reported under atypical genetic conditions, such as skewed X-inactivation or chromosomal rearrangements (4, 5).

There is a growing interest in how environmental factors may influence the expression and severity of MD. Among these, maternal micronutrient balance during pregnancy deserves attention—particularly the potential role of excessive zinc supplementation (6). Zinc and copper are antagonistic trace elements, and high levels of zinc can impair copper absorption by inducing intestinal metallothionein, which preferentially binds copper and prevents its systemic availability (7).

Zinc is a vital micronutrient required for immune regulation, enzymatic activity, and fetal development. During pregnancy, zinc supplementation is frequently recommended to enhance maternal immunity, reduce the risk of infections, and support overall gestational health (8). It is often included in prenatal vitamins and sometimes prescribed in higher doses, especially in settings where micronutrient deficiencies are common. However, excessive zinc intake—particularly if not medically supervised—can disrupt copper homeostasis, potentially leading to secondary copper deficiency and related complications (9–11).

We propose a mechanistically grounded but unverified hypothesis that excessive maternal zinc intake during pregnancy may influence fetal copper homeostasis in cases of ATP7A mutation, thereby potentially modifying the phenotypic expression of Menkes disease. However, direct clinical evidence remains limited, with only a small number of studies in pregnant women demonstrating an inverse relationship between zinc supplementation and copper status and no large-scale epidemiological data linking this interaction to neonatal outcomes.

## Copper requirements in humans: physiological role and clinical relevance

Copper is a crucial trace element essential for various physiological and biochemical functions in the human body. It is involved in numerous enzymatic processes that support energy metabolism, antioxidant defence, nervous system function, iron transport, and connective tissue formation (7, 12, 13). Enzymes that rely on copper include cytochrome c oxidase for mitochondrial respiration (14), superoxide dismutase for neutralizing reactive oxygen species (15, 16), lysyl oxidase for collagen and elastin crosslinking (17), and ceruloplasmin for iron mobilization (18, 19). In addition, copper-dependent enzymes like dopamine *β*-hydroxylase are key for neurotransmitter synthesis, linking copper to neurodevelopmental health (20). These wide-ranging roles highlight the necessity of maintaining optimal copper levels, particularly during pregnancy and infancy, when cellular growth and differentiation are at their peak.

**Figure 1:**
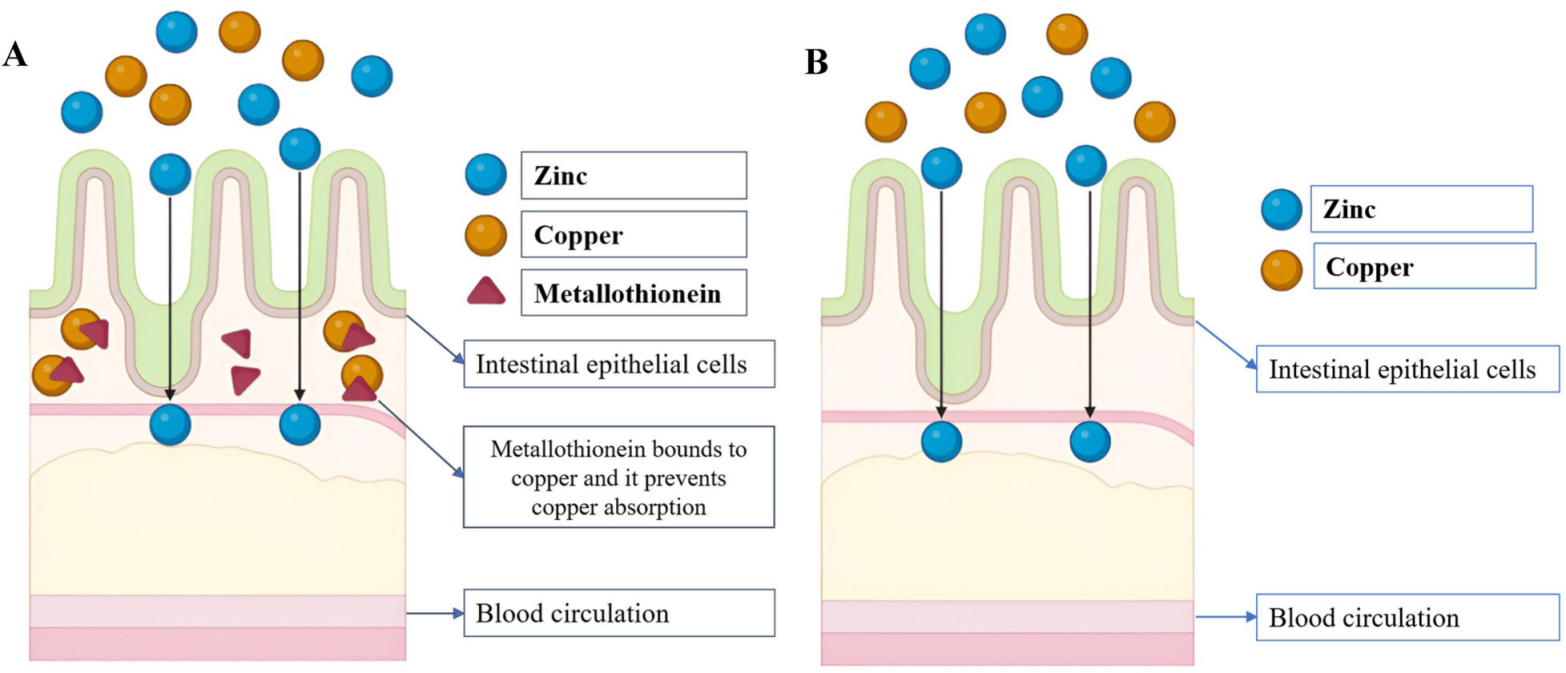
**(A)** zinc excess state can cause overexpression of metallothionein, preventing copper absorption; **(B)** zinc excess state can directly impair copper absorption.

Copper is mainly absorbed in the upper small intestine, especially the duodenum, through copper transporter proteins like CTR1. Once absorbed, it binds to proteins such as albumin and is delivered to the liver, where it is incorporated into ceruloplasmin for systemic circulation. Copper excretion occurs primarily through bile (21). However, this tightly regulated system can be disrupted by nutritional imbalances, particularly through zinc-induced metallothionein expression, which reduces copper bioavailability (22). As a result, excessive zinc consumption can indirectly cause copper deficiency even if copper intake remains adequate, which is depicted in Figure 1(A). Moreover, emerging evidence suggests that elevated luminal zinc concentrations can directly impair copper entry into intestinal epithelial cells, independent of MT-mediated sequestration, leading to systemic copper deficiency, which is depicted in Figure 1(B).

When copper levels fall below normal, multiple organ systems can be affected. Neurological symptoms such as muscle weakness, irritability, delayed motor development, and seizures may arise due to impaired myelination and neurotransmitter activity (23). Hematologic manifestations like anemia and neutropenia are also common because of copper's role in iron transport and bone marrow function (24). Moreover, connective tissue defects, bone abnormalities, pale skin or hair, and poor immune responses can occur, reflecting the systemic impact of copper-dependent enzymes. Infants and neonates are particularly vulnerable, and in severe cases, copper deficiency can mimic neurodegenerative or metabolic disorders.

Copper is found in many everyday foods, including organ meats (especially beef liver), Shellfish (e.g., oysters, crabs), Nuts and seeds (cashews, sunflower seeds), Whole grains, Dark chocolate, Legumes (lentils, chickpeas). High amount of copper is found in Oysters (44,996 µg per 100 g), beef liver (6,434 µg per 100 g) and in cocoa (5000 µg per 100 g). Breast milk also supplies sufficient copper in early infancy, although its concentration decreases over time (25–27) Infants with gastrointestinal disorders or those dependent on parenteral nutrition may face a higher risk of deficiency. Furthermore, inherited conditions like Menkes disease—a disorder caused by mutations in the ATP7A gene, which disrupts copper absorption and transport—can result in severe systemic copper deficiency despite normal dietary intake. This condition underscores the importance of both nutrient intake and the body's ability to properly utilize essential trace elements (28).

## Role of copper in fetal and neonatal health

The body's copper requirements vary with age and physiological status. As outlined by the National Institutes of Health (2021), Infants need about 200 µg/day during the first six months, while adults require approximately 900 µg/day. These values increase during pregnancy (1000 µg/day) and lactation (1,300 µg/day) to support fetal growth and neonatal development (29). During the third trimester, copper transfer from the mother to the fetus intensifies, as this is the critical period when the fetus builds hepatic copper reserves needed after birth. Thus, maternal nutritional status directly affects fetal copper supply and postnatal copper sufficiency (30).

During pregnancy, the fetus is entirely dependent on maternal copper for establishing hepatic copper stores, which are crucial for early postnatal life, especially in preterm infants who have reduced storage capacity. Copper plays a fundamental role in neurodevelopment, influencing brain growth, myelination, neurotransmitter synthesis, and synaptic function. Deficiencies during gestation have been linked to impaired central nervous system development, structural abnormalities, and long-term cognitive deficits (31). Additionally, copper is essential for haematopoiesis, participating in iron metabolism via ceruloplasmin activity, which aids in iron mobilization and incorporation into haemoglobin (32).

Copper plays a critical role in angiogenesis, the formation of new blood vessels, a process indispensable for proper placental function and fetal organogenesis (33). Copper-dependent enzymes such as lysyl oxidase contribute to the stabilization of the extracellular matrix by catalysing the cross-linking of collagen and elastin fibers. This enzymatic activity ensures the structural integrity and elasticity of developing tissues and vascular networks. Inadequate copper impairs endothelial cell proliferation and disrupts the remodelling of connective tissue, potentially compromising placental vascularization, which is essential for efficient maternal-fetal nutrient and oxygen exchange (34, 35).

Beyond structural roles, copper is fundamental in shaping the neonatal immune system. Copper-dependent enzymes, including ceruloplasmin and Cu/Zn superoxide dismutase, protect against oxidative stress and assist in the maturation and function of immune cells (36–38) Copper deficiency has been shown to impair neutrophil bactericidal activity and reduce T-lymphocyte proliferation, leading to heightened susceptibility to infections in neonates and infants (39). These immunological impairments underscore the importance of maintaining adequate copper levels during fetal development and early postnatal life.

The transfer of copper from mother to fetus is a tightly regulated process, occurring predominantly in the third trimester of pregnancy, a critical window when the fetus builds hepatic copper stores for postnatal use. This transfer is mediated by copper-transporting ATPases, primarily ATP7A and ATP7B, expressed in the placenta. These transporters regulate the movement of copper across trophoblast layers into fetal circulation. Disruptions in transporter function—whether due to genetic mutations (as in Menkes disease) or secondary to maternal copper deficiency—can result in severely diminished fetal copper levels (40).

Consequently, inadequate maternal copper status or placental dysfunction may lead to a spectrum of neonatal complications, including intrauterine growth restriction (IUGR), bone fragility, hypopigmentation, anemia, impaired thermoregulation, and early-onset neurological deficits (41, 42). These adverse outcomes highlight the direct dependency of fetal and neonatal copper homeostasis on maternal copper availability, reinforcing the need to monitor and optimize maternal trace element nutrition during pregnancy for favourable developmental outcomes.

## Placental regulation of copper and zinc transport

Copper transport across the placenta is tightly regulated by coordinated action of CTR1 for cellular uptake and ATP7A for export into fetal circulation. Zinc transport is mediated by ZIP (import) and ZnT (export) transporters. Elevated maternal zinc levels upregulate placental metallothionein expression, which has a higher binding affinity for copper, potentially sequestering copper within placental cells and limiting its transfer to the fetus. In addition, competitive interactions at shared transport pathways may further impair copper translocation. Although these mechanisms are biologically plausible, direct human experimental validation remains limited.

## Zinc supplementation in pregnancy: benefits versus risks

Zinc is essential for various physiological functions, including immunity, growth, neurological activity, vision, and reproductive health. During pregnancy, adequate zinc intake is advised as it may help lower the risk of preeclampsia, preterm delivery, and infantile asthma. Two meta-analyses, each comprising 14 studies, demonstrated that low maternal serum zinc concentrations are significantly linked to an increased risk of pregnancy-induced hypertension (PIH) and preeclampsia (43, 44). Furthermore, maternal zinc deficiency has been inversely associated with the occurrence of asthma in children by the age of five (45).

In a study of 450 pregnancies, women with zinc levels in the lowest quartile experienced the highest incidence of both maternal and fetal complications, particularly fetal distress (44). A meta-analysis of 16 randomized controlled trials reported a 14% reduction in preterm births following zinc supplementation, although no other significant maternal or neonatal benefits were observed (46). The majority of these trials administered 20–30 mg of zinc, predominantly in the form of zinc sulfate. In another study, a daily dose of 25 mg of zinc sulfate given to women with relatively low plasma zinc levels in early pregnancy was linked to increased birth weights and head circumferences in infants (47). However, zinc supplement showed no improvement in neurological development test scores of the infants (48).

A study reported that daily supplementation of 30 mg of zinc in pregnant women led to a 54% decrease in the likelihood of impetigo—a bacterial skin infection—in their infants (49). In addition to zinc sulfate, preliminary evidence suggests that zinc lactate may also be effective. One such study evaluated doses of 0, 5, 10, and 30 mg of zinc lactate found that the 30 mg/day dose was associated with notable reductions in the incidence of low birth weight, preterm delivery, and intrauterine growth restriction (50). Another comprehensive review compared findings from various studies on zinc supplementation and recommended a daily prenatal dose of 30 mg, which can decrease the risk of preterm birth and may also help lower the chances of impetigo, asthma, and preeclampsia. However, the authors highlighted the need for further research to confirm these potential benefits (51).

Due to these benefits, zinc supplements are widely prescribed or self-administered during pregnancy, especially in low- and middle-income countries where maternal micronutrient deficiencies are prevalent. Zinc is also marketed as a general immune booster, and during periods of heightened infection risk—such as the COVID-19 pandemic—its use surged, sometimes beyond recommended levels, including among pregnant women. In such contexts, copper-zinc interaction becomes concerning, because the fetus is entirely reliant on maternal copper stores for its own development.

## Evidence of zinc-induced copper deficiency

The antagonistic relationship between zinc and copper has been well-documented in both experimental and clinical settings. Numerous studies have demonstrated that excessive zinc intake disrupts copper homeostasis through mechanisms described earlier, including metallothionein-mediated sequestration, leading to copper entrapment and subsequent fecal excretion (22). One of the earliest human reports of this phenomenon was observed in individuals receiving high-dose zinc therapy for Wilson's disease or acne, where prolonged zinc administration led to hypocupremia, neutropenia, and anemia (52).

In a case study of a 57-year-old woman with a past medical history of end-stage renal disease, had been receiving excess zinc for several months and subsequently experienced erythropoietin resistant anemia. After bone marrow biopsy and more serological testing, she was ultimately diagnosed with Zinc-induced copper deficiency (ZICD). After cessation of her zinc supplement and initiation of copper replacement proved effective in restoring erythropoietin responsiveness. This case highlights zinc toxicity as a cause of secondary copper deficiency anemia. And they recommended Periodic screening of zinc and copper levels in patients who received prolonged zinc supplementation and have developed unexplained anemia (53, 54).

Another study treated rats with high dietary or high parenteral Zinc to study copper metabolism. In zinc treated rats, low serum copper concentration, low serum ceruloplasmin activity and low cytochrome C oxidase activity was recorded. None of these changes, were related to zinc induced metallothionein in the intestinal epithelial cell. This study suggests that the effects of high zinc treatment on copper status are not the result of the long-held theory that zinc induced intestinal metallothionein sequesters copper and prevents its passage to the circulation. Instead, it may be caused by a direct effect of high luminal zinc concentrations on copper transport into the epithelial cell (22).

Multiple clinical reports have demonstrated that prolonged or excessive zinc supplementation can result in severe copper deficiency, manifesting as anemia, neutropenia, and neurological complications such as myelopathy, peripheral neuropathy, and leukoencephalopathy (22, 55–61). These effects have been observed both in the context of medically prescribed zinc therapy, such as in Wilson disease, and through unregulated or over-the-counter zinc supplement use. Even standard doses of zinc, when used chronically, have been implicated in copper deficiency, underscoring the delicate balance between these trace elements (62, 63).

Recent studies have also raised concern regarding the effects of zinc supplementation during pregnancy. It has been shown that maternal zinc intake significantly reduces both maternal and umbilical cord copper levels (64). Given the fetus's complete reliance on maternal copper supply, any impairment in copper transfer may increase the risk of fetal copper deficiency. This concern is especially relevant in pregnancies where the fetus carries ATP7A mutations, as even marginal disruptions in copper availability could exacerbate disease severity or precipitate early onset of MD. In a double-blind cross-over trial lasting 12 weeks trial, healthy adults receiving 150 mg/day of elemental zinc for six weeks experienced a significant decline in serum copper levels and ceruloplasmin activity, confirming zinc's role in disrupting copper homeostasis even in the absence of overt copper deficiency (65). While the inhibitory effect of high zinc on copper absorption is well documented in adult populations, no large-scale clinical studies or birth cohort analyses have systematically investigated this interaction in the context of fetal copper homeostasis.

## Dose-Dependent effects of zinc: physiological versus excess exposure

Interpretation of zinc-related effects requires clear distinction between physiological and excessive exposure. The recommended dietary allowance (RDA) during pregnancy is approximately 11–12 mg/day, while the tolerable upper intake level (UL) is 40 mg/day. Most evidence of zinc-induced copper deficiency arises from chronic intake exceeding the UL or pharmacological dosing (e.g., ≥100–150 mg/day). Therefore, adverse copper interactions are unlikely under standard dietary conditions but may become relevant in settings of high-dose or prolonged unsupervised supplementation, particularly in vulnerable populations.

## Menkes disease: genetics, phenotype, and potential environmental modifiers

Mutations in the ATP7A gene code for a rare X-linked neurological disease called Menkes disease (MD). This gene is essential for copper transport across cell membrane. Defective copper transport brought on by this mutation can result in a copper deficiency that causes severe neurological degeneration, abnormalities of connective tissue, and the distinctive brittle, steel-coloured hair that affected infant's display. Clinical symptoms typically appear within the first few months of life, and without timely intervention, most children with MD succumb within three years (1). While MD is fundamentally genetic, the phenotypic severity and timing of symptom onset may be influenced by environmental modifiers.

One such potential modifier is maternal micronutrient status during pregnancy. Given the competitive interaction between zinc and copper at the level of intestinal and placental absorption, excess maternal zinc supplementation may further impair copper transfer to the fetus, particularly in individuals harbouring ATP7A mutations. This additional reduction in copper availability may exacerbate the onset and severity of MD symptoms, including more rapid neurodegeneration or earlier-onset connective tissue pathology. Although this hypothesis has yet to be thoroughly examined, it introduces a compelling gene–environment interaction model where excess zinc intake could worsen the biochemical consequences of ATP7A dysfunction. Identifying such modifiers may offer new insights into disease variability and support the refinement of prenatal care recommendations, especially regarding trace mineral supplementation in genetically susceptible populations.

## Preclinical evidence of maternal high-zinc exposure inducing copper deficiency in neonates

Experimental animal models provide strong evidence for zinc–copper antagonism during pregnancy and early life. In sheep, high maternal zinc intake (750 mg/kg diet) induced pronounced copper deficiency, resulting in abortions, stillbirths, and impaired maternal growth and feed efficiency. Notably, while copper supplementation corrected biochemical deficiency, it failed to fully restore growth and offspring viability, indicating additional zinc-related toxicity (66).

Consistent findings are observed in pig models, where maternal exposure to very high zinc levels (5,000 ppm) precipitated rapid copper deficiency in neonates when dietary copper was inadequate. This was characterized by growth retardation, anemia, reduced ceruloplasmin activity, diminished copper-dependent enzymes, and depleted tissue copper store (67). Importantly, these effects were largely reversed with modest copper supplementation. Together, these studies demonstrate that excessive zinc disrupts copper homeostasis across maternal and early developmental stages, supporting the biological plausibility of zinc-induced copper deficiency and its adverse developmental consequences. However, much of the available evidence is derived from adult populations, animal models, or pharmacological zinc exposure, which may not fully reflect physiological conditions during pregnancy.

## Clinical evidence of zinc–copper antagonism during pregnancy

Clinical data in pregnant women demonstrated a measurable inverse relationship between zinc supplementation and copper status. In a controlled clinical study, maternal serum copper in the untreated control group increased physiologically from 117.15 ± 2.12 µg/dL (first trimester) to 138.57 ± 0.92 µg/dL (third trimester; *p* < 0.001). In contrast, women receiving zinc (∼45 mg/day) showed a significant decline in serum copper from 115.64 ± 1.12 µg/dL to 111.10 ± 0.99 µg/dL (*p* < 0.001) over the same period. Notably, fetal exposure was also influenced: the zinc-supplemented group exhibited substantially lower umbilical cord serum copper levels than the controls (*p* < 0.05), with the extent of the reduction being directly proportional to the duration of zinc therapy (64). Importantly, this remains one of the very few clinical studies—and to our knowledge, the only available evidence—demonstrating reduced fetal copper status, as reflected by umbilical cord serum copper levels, in neonates born to mothers receiving zinc supplementation compared to those without zinc exposure.

## Clinical and epidemiological gaps and recommendations

Despite the well-established genetic basis of Menkes disease, important gaps remain in understanding how maternal trace element balance influences fetal copper status. Emerging clinical evidence demonstrates that zinc supplementation during pregnancy can significantly reduce both maternal and fetal copper levels, with umbilical cord copper concentrations decreasing in proportion to the duration of zinc exposure (*p* < 0.05). However, these findings have not yet been translated into population-level insights. Thus, while emerging clinical data support biological plausibility, the hypothesis remains preliminary and requires targeted validation.

From an epidemiological perspective, no large-scale studies have stratified neonatal copper status or Menkes disease presentation based on maternal zinc intake patterns. This represents a critical blind spot in identifying potentially modifiable risk factors that may influence disease severity or early onset. Additionally, retrospective analyses linking maternal supplementation practices with neonatal copper-related outcomes—particularly in infants with undiagnosed or subclinical ATP7A dysfunction—are lacking.

**Figure 2:**
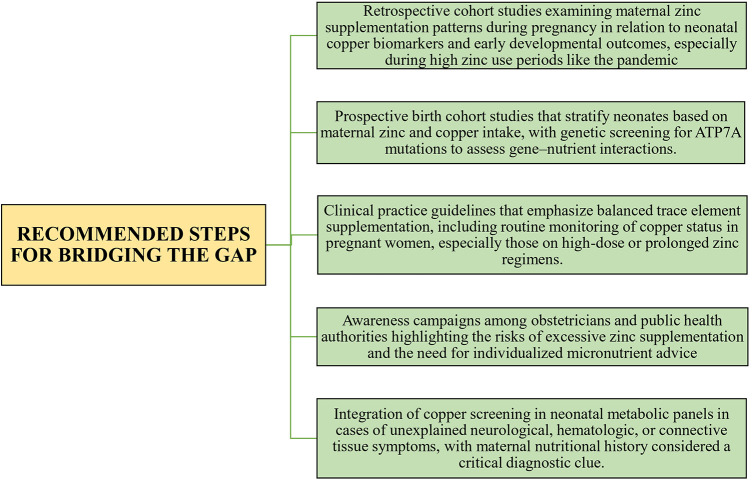
Recommended strategies for bridging research and clinical gaps in maternal zinc exposure and neonatal copper deficiency disorders.

To address these gaps, systematic epidemiological surveillance, retrospective cohort analyses, and prospective studies evaluating maternal zinc exposure in relation to fetal copper status are urgently needed. Key recommendations are outlined in Figure 2.

## Conclusion

Copper is a critical trace element for fetal development, with the fetus relying heavily on maternal supply, particularly in late gestation. Emerging clinical evidence demonstrates that maternal zinc supplementation can reduce both maternal and fetal copper levels, highlighting a biologically significant antagonistic interaction. While Menkes disease is primarily driven by ATP7A mutations, this review proposes that excessive zinc exposure during pregnancy may act as an environmental modifier, further impairing copper transfer and potentially exacerbating disease severity in susceptible fetuses. Despite this plausible link, current guidelines overlook zinc–copper interactions, and epidemiological evidence remains limited. We therefore advocate for targeted retrospective analyses of maternal zinc use and well-designed prospective studies integrating maternal–fetal micronutrient assessment. Addressing these gaps could enhance early diagnosis, improve risk stratification, and inform safer, evidence-based supplementation strategies in pregnancy.
